# Evaluation of 3D Markerless Motion Capture Accuracy Using OpenPose With Multiple Video Cameras

**DOI:** 10.3389/fspor.2020.00050

**Published:** 2020-05-27

**Authors:** Nobuyasu Nakano, Tetsuro Sakura, Kazuhiro Ueda, Leon Omura, Arata Kimura, Yoichi Iino, Senshi Fukashiro, Shinsuke Yoshioka

**Affiliations:** ^1^Department of Life Sciences, Graduate School of Arts and Sciences, The University of Tokyo, Tokyo, Japan; ^2^Research Fellow of the Japan Society for the Promotion of Science, Tokyo, Japan; ^3^Department of General Systems Studies, Graduate School of Arts and Sciences, The University of Tokyo, Tokyo, Japan

**Keywords:** openPose, markerless, motion capture, biomechanics, human movement

## Abstract

There is a need within human movement sciences for a markerless motion capture system, which is easy to use and sufficiently accurate to evaluate motor performance. This study aims to develop a 3D markerless motion capture technique, using OpenPose with multiple synchronized video cameras, and examine its accuracy in comparison with optical marker-based motion capture. Participants performed three motor tasks (walking, countermovement jumping, and ball throwing), and these movements measured using both marker-based optical motion capture and OpenPose-based markerless motion capture. The differences in corresponding joint positions, estimated from the two different methods throughout the analysis, were presented as a mean absolute error (MAE). The results demonstrated that, qualitatively, 3D pose estimation using markerless motion capture could correctly reproduce the movements of participants. Quantitatively, of all the mean absolute errors calculated, approximately 47% were <20 mm, and 80% were <30 mm. However, 10% were >40 mm. The primary reason for mean absolute errors exceeding 40 mm was that OpenPose failed to track the participant's pose in 2D images owing to failures, such as recognition of an object as a human body segment or replacing one segment with another depending on the image of each frame. In conclusion, this study demonstrates that, if an algorithm that corrects all apparently wrong tracking can be incorporated into the system, OpenPose-based markerless motion capture can be used for human movement science with an accuracy of 30 mm or less.

## Introduction

Motion capture systems have been used extensively as a fundamental technology within biomechanics research. However, traditional marker-based approaches have significant environmental constraints. For example, measurements cannot be performed in environments wherein wearing markers during the activity is difficult (such as sporting games). Markerless measurements without such environmental constraints can facilitate new understanding about human movements (Mündermann et al., 2006); however, complex information processing technology is required to make an algorithm that recognizes human poses or skeletons from images. Therefore, it is desirable to many biomechanics researchers to develop a markerless motion capture that is easy to use for measurement.

Recently, automatic human pose estimation using deep learning techniques have attracted attention amongst computer vision researchers. Most of these algorithms train the neural network using manually labeled image data and then estimate the human posture, such as joint centers and skeletons, when the user inputs the images or videos to the trained network. When compared to the approach using RGB-Depth cameras such as Kinect (Clark et al., 2012; Pfister et al., 2014; Schmitz et al., 2014; Gao et al., 2015), a deep-learning-based approach has less constraints on the distance between the camera and the target to be measured as well as the sampling rate of video recording. Deep-learning-based approaches have begun with 2D pose estimation, which automatically estimates human joint centers from 2D RGB images, outputting the 2D coordinates in the images (Toshev and Szegedy, 2014; Wei et al., 2016; Papandreou et al., 2018). Additionally, it is possible to identify the 3D human joint locations in the global coordinate system using the 2D human joint locations in synchronized multi-view video camera images following the same procedure as a marker-based motion capture system. More recently, deep-learning-based computer vision studies have been working on 3D pose estimation, which estimates the 3D human joint locations directly using a single algorithm. There have been studies of 3D pose estimation using a single-view camera image (Chen and Ramanan, 2017; Pavlakos et al., 2018; Moon et al., 2019) or multi-view camera images (Rhodin et al., 2018; Iskakov et al., 2019; Pavllo et al., 2019) as input of the pose estimation algorithm.

However, biomechanics researchers require ease of use and sufficient accuracy to achieve the aims of motion analysis. Seethapathi et al. (2019) reviewed pose tracking studies from the perspective of movement science and pointed out that deep-learning-based human pose tracking algorithms did not prioritize the quantities that matter for movement science. It remains unclear whether the accuracy of the deep-learning-based 3D markerless motion capture is appropriate for human movement studies such as sports biomechanics or clinical biomechanics.

Although computer-vision researchers are working to improve the deep-learning-based pose estimation algorithms (e.g., calculation speed and/or correct tracking rate), development of these algorithms is outside the scope of general biomechanics research that investigate the functional mechanisms, injury prevention, rehabilitation, and motor control of human movements. Thus, biomechanics researchers should utilize a combination of publicly available deep-learning-based software and the principles of conventional motion capture systems such as camera calibration or kinematic data processing techniques. OpenPose is one of the most popular open-source pose estimation technologies (Cao et al., 2018) and is deemed easy to use for biomechanics researchers. Therefore, the aim of this study was to develop a 3D markerless motion capture using OpenPose with multiple synchronized video cameras and then assess the accuracy of the 3D markerless motion capture by comparing with an optical marker-based motion capture.

## Materials and Methods

### Participants

Two healthy male volunteers participated in this experiment. The mean age, height, and body mass of the participants were 22.0 years, 173.5 cm, and 69.5 kg, respectively. The participants provided written informed consent prior to the commencement of the study, and the experimental procedure used in this study was approved by the Ethics Committee of the university with which the authors were affiliated.

### Overview of Data Collection

Participants performed three motor tasks in the order of walking, countermovement jumping, and ball throwing. These motor tasks were chosen to include different levels of degrees of freedom and speeds of movement; a walking task is a 2D slow movement, a jumping task is a 2D quick movement, and a throwing task is a 3D quick movement. These movements were measured using both a marker-based optical motion capture and a video camera-based (markerless) motion capture. A light was used to synchronize the data obtained from all the video cameras and the two different measurement systems as follows. A light turned on at the center of the measurement space when it was switched on by an experimenter before and after a single trial. The switching on of the light before and after a single trial was recorded by each video camera. The light switch-on frame in a video was manually detected for each camera using Adobe Premiere Pro (Adobe Inc, San Jose, CA, USA). The synchronization frame was determined as one of the start or end frames, depending on the trial, because sometimes one of the start or end frames could not be seen from all camera views. After the synchronization frame was determined, the total number of frames for each video camera was set to be equal. In addition, the light switch-on frame in the analog signals for the marker-based optical motion capture was detected. Finally, marker-based motion capture data was down-sampled to the same sampling frequency as that of the markerless motion capture data.

### Marker-Based Motion Capture

Forty-eight reflective markers were attached onto body landmarks, as described in our previous study (Kimura et al., 2019). The coordinates of these reflective markers upon the participants' bodies were recorded using a 16-camera motion capture system (Motion Analysis Corp, Santa Rosa, CA, USA) at a sampling rate of 200 Hz. The elbow, wrist, knee, and ankle joint centers were assigned to the mid-points of the lateral and medial markers, while the shoulder joint centers were assigned to the mid-points of the anterior and posterior shoulder markers. The hip joint centers were estimated using the method described by Harrington et al. (2007). The raw kinematic data was smoothed using a zero-lag fourth order Butterworth low-pass filter. The cut-off frequency of the filter was determined using a residual analysis (Winter, 2009). Data analysis was performed using MATLAB (v2019a, MathWorks, Inc., Natick, MA, USA).

### Markerless Motion Capture

**Figure 1:**
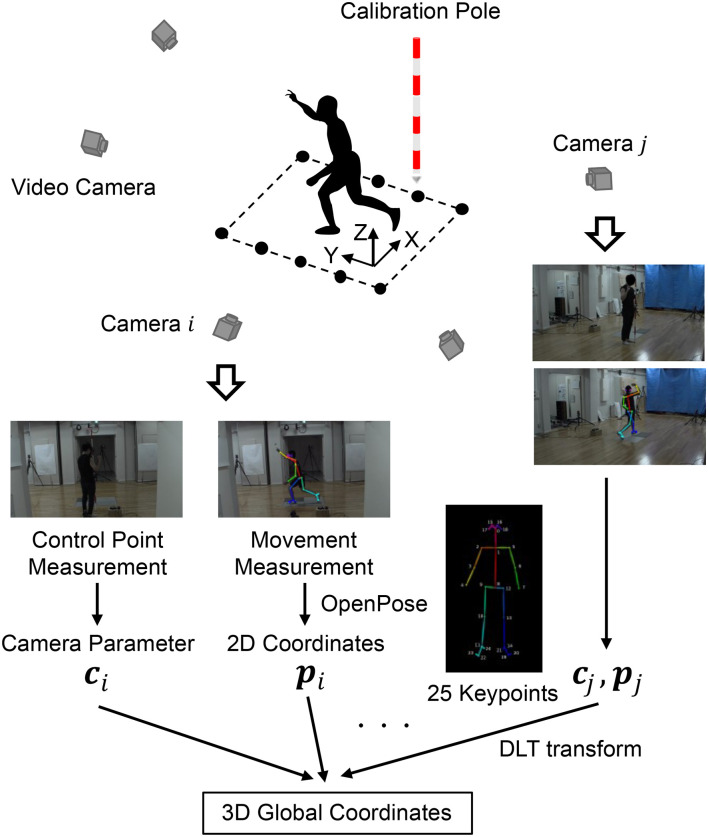
Experimental setup and overview of the markerless motion capture.

The experimental setup and overview of the markerless motion capture are shown in Figure 1. The definition of the coordinate system is as follows that X is lateral/medial, Y is anterior/posterior, and Z is inferior/superior. The markerless motion capture consisted of five video cameras (GZ-RY980, JVCKENWOOD Corp, Yokohama, Kanagawa, Japan). Two measurement conditions, i.e., combinations of video camera resolutions and sampling frequencies, were implemented: 1, 920 × 1, 080 pixels at 120 Hz (1K condition) and 3, 840 × 2, 160 pixels at 30 Hz (4K condition). OpenPose (version 1.4.0) was installed from GitHub (CMU-Perceptual-Computing-Lab, 2017) and run with GPU (GEFORCE RTX 2080 Ti, Nvidia Corp, Santa Clara, CA, USA) under default settings. Twenty-five keypoints (Figure 1) of the participant's body were outputted independently for each frame via OpenPose execution (for details, see CMU-Perceptual-Computing-Lab, 2017). The control points, at which 3D global coordinates could be identified, were measured using the video cameras with use of a calibration pole. The 2D video camera coordinates obtained from OpenPose were transformed to 3D global coordinates using a direct linear transformation (DLT) method (Miller et al., 1980). The raw kinematic data was smoothed using a zero-lag fourth order Butterworth low-pass filter. The cut-off frequency of the filter was determined using residual analysis (Winter, 2009), and the ranges were 5–8 and 2–3 Hz in the 1K and 4K conditions, respectively.

### Data Analysis

The position data obtained using the marker-based motion capture was downsampled using the spline function to alter the number of frames such that they were the same as those obtained using markerless motion capture. The analysis period durations were defined for each individual motor task: from the second step heel contact to the next heel contact of the same leg in a walking task, from the start of the squatting motion to the recovery of the initial upright stance in a jumping task, and from the toe-off on the opposite side of the throwing arm to the end of the arm-swing in a throwing task. The differences in the corresponding joint positions that were estimated from the two different motion captures throughout the analysis durations were calculated. Mean absolute error (MAE) of the two time-series data during the analysis period durations was used as the indicator of the difference as described by Equation (1), where, *n* is the number of frames, *x*_*m*_ and *x*_*o*_ are the positions estimated by the marker-based and OpenPose-based approaches, respectively. It should be noted that the positions of the landmarks that are tracked by OpenPose do not necessarily correspond to the points estimated by the marker-based approach. Therefore, to evaluate the accuracy of markerless motion capture, we compared the corresponding positions of the shoulder, elbow, wrist, hip, knee, and ankle joints, which can be estimated by two motion captures.

## Results

**Figure 2:**
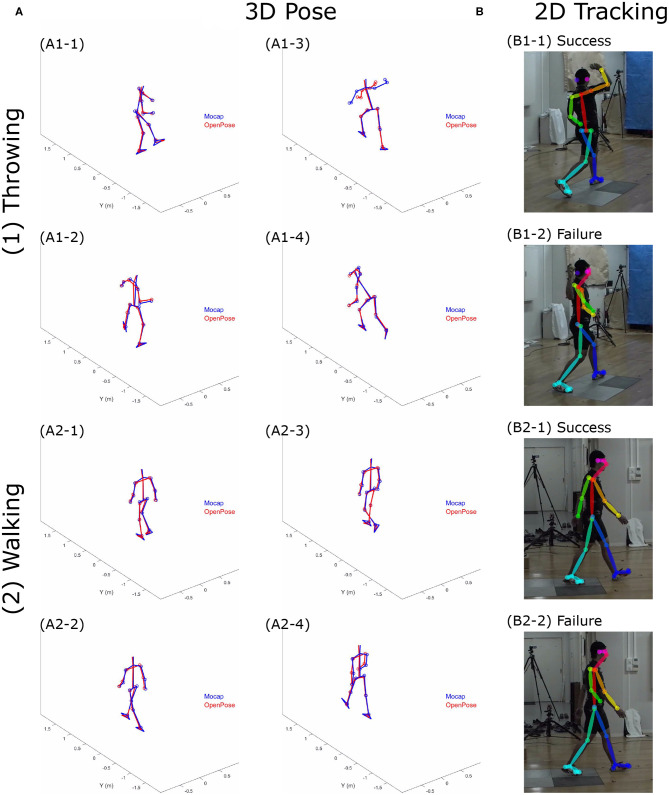
**(A)** Examples of participant's pose estimated by the marker-based motion capture (Mocap) and by the OpenPose-based markerless motion capture (OpenPose); **(B)** Examples of 2D pose tracking success and failure during a (1) ball throwing task and (2) walking task. We defined the apparently incorrect position tracking as failures and defined the others as success based on our visual inspection.

**Figure 3:**
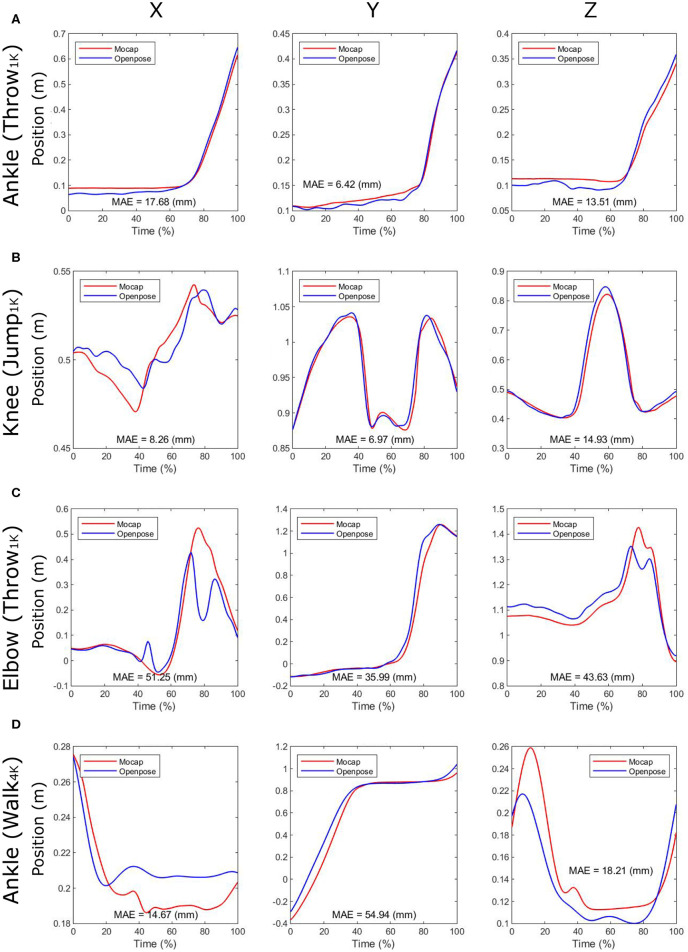
Time series profiles of joint positions estimated by the marker-based motion capture (Mocap) and by the markerless motion capture using OpenPose (OpenPose). Here, the X, Y, and Z positions of **(A)** the ankle joint for throwing under the 1K condition, **(B)** the knee joint for jumping under the 1K condition, **(C)** the elbow joint for throwing under the 1K condition, and **(D)** the ankle joint for walking under the 4K condition are shown as representative plots. The mean absolute error (MAE) through analysis duration is shown in each panel. Also, _1*K*_ and _4*K*_ represent that the task was recorded under 1K (1, 920 × 1, 080 pixels at 120 Hz) and 4K (3, 840 × 2, 160 pixels at 30 Hz) conditions when using video-camera-based motion capture, respectively. X is lateral/medial, Y is anterior/posterior, and Z is inferior/superior direction.

Examples of the 3D pose estimations obtained by the two different motion captures are depicted in Figure 2A. In addition, video examples that show the participant's pose during movements are provided as Supplementary Materials to this paper. The representative time-series profiles of joint positions estimated by both the marker-based motion capture (Mocap) and the OpenPose-based markerless motion capture (OpenPose) can be seen in Figure 3. Here, the X, Y, and Z positions of the ankle joint for throwing under the 1K condition, the knee joint for jumping under the 1K condition, the elbow joint for throwing under the 1K condition, and the ankle joint for walking under the 4K condition are shown as representative plots. The MAE of the two plots throughout the duration of analysis is shown in each panel. Qualitatively, the shapes of the time-series profiles were found to be approximately the same.

Quantitatively, the MAE of joint positions in Figures 3A,B were <20 mm; however, the MAEs of joint positions in Figures 3C,D were >40 mm. The absolute errors were particularly large at specific moments within the analysis duration (i.e., approximately 45 and 80% of the time in Figure 3C, as described in Figures 2A1–3). The MAEs of the corresponding joint positions, estimated from the two different motion captures for all trials, are presented in Table 1. Of these MAEs in Table 1, approximately 47% are <20 mm, 80% are <30 mm, and 10% are >40 mm.

**Table 1:** The MAEs (mm) as the differences of corresponding joint positions estimated from the two different motion captures.

		**Walk_1K_**	**Walk_4K_**	**Jump_1K_**	**Jump_4K_**	**Throw_1K_**	**Throw_4K_**
	X	28.4	24.6	27.1	20.9	21.7	19.3
ShoulderR	Y	17.0	49.7	12.5	11.9	27.7	32.5
	Z	17.9	16.5	29.5	30.8	15.8	13.9
	X	4.32	6.96	9.36	8.25	47.3	45.2
ElbowR	Y	37.0	66.8	27.0	20.5	28.7	35.1
	Z	21.7	22.2	26.9	35.3	38.0	38.9
	X	5.78	7.52	8.96	8.31	40.6	47.5
WristR	Y	19.0	44.2	13.2	20.6	28.7	40.3
	Z	15.7	16.8	23.8	38.9	24.7	26.7
	X	9.65	7.67	8.77	6.01	29.5	25.0
HipR	Y	21.3	49.4	15.0	14.2	13.5	20.8
	Z	24.4	20.6	31.0	32.1	27.2	23.5
	X	6.41	4.09	7.74	6.47	15.2	13.1
KneeR	Y	25.9	48.2	8.48	18.3	13.8	19.1
	Z	10.1	11.4	14.8	20.9	24.4	20.4
	X	9.68	8.73	9.82	6.67	12.3	19.1
AnkleR	Y	28.6	58.1	9.31	11.0	17.7	22.2
	Z	11.7	20.7	20.6	27.9	12.4	20.3

*Also, _1K_ and _4K_ represent that the task was recorded under 1K (1, 920 × 1, 080 pixels at 120 Hz) and 4K (3, 840 × 2, 160 pixels at 30 Hz) conditions when using video-camera-based motion capture, respectively. X is lateral/medial, Y is anterior/posterior, and Z is inferior/superior direction*.

The accuracy of the 3D pose estimation using the markerless motion capture depends on 2D pose tracking by OpenPose. Because the algorithm that tracks the human pose was applied to each frame of the video independently, within a single trial, there are frames where the participant's pose was well tracked, whereas in others the participant's pose was not well tracked. Examples of pose estimation successes and failures using OpenPose are depicted in Figure 2B. Based on our visual inspection, we defined the apparently incorrect position tracking as failures and the remaining as successes.

## Discussion

This study aimed to examine the accuracy of 3D markerless motion capture using OpenPose with multiple video cameras through comparison with an optical marker-based motion capture. Qualitatively, 3D pose estimation using the markerless motion capture approach can correctly reproduce the movements of participants (Figure 2A and Supplementary Videos). The MAE in 80% of all trials conducted was found to be <30 mm. This relatively small error may be due to the shortcomings in the OpenPose tracking precision. Because the majority of computer vision-based pose tracking algorithms, including OpenPose, are based on the supervised learning using manually labeled data, it is inevitable that small errors in 3D pose are caused by inherent noise in the training data.

Relatively large MAEs exceeding 40 mm were observed in certain cases (Figure 3). Observing the estimated pose during movements reveals that the correct joint positions (including noises) are estimated in the trial, including a relatively small error that is <30 mm (e.g., Figures 2A1-1, A1-2, A1-4); however, apparently incorrect joint positions are estimated in the trial including relatively large errors that are more than 40 mm (*e.g.*, Figure 2A1-3). The primary reason for estimating apparently incorrect 3D positions is that OpenPose failed to track the participant's pose depending on the image of each individual frame (Figure 2B). Due to the 2D tracking failures, correction of such failures is required to achieve a more accurate 3D pose estimation. Within this study, for example, the interchange of the left and right segments was retrospectively corrected because, without this correction, the 3D pose was completely different from the human shape. However, recognizing an object as a human body segment (e.g., failures in Figure 2B) was not corrected because it may have required manual tracking; moreover, without the correction, the 3D pose accuracy could be evaluated. Therefore, to use the OpenPose-based markerless motion capture in human movement science studies, it is considered necessary to incorporate algorithms that can correct all such tracking failures.

There are several methods for fixing the tracking failures. One approach is to use the same procedure as that used in the software to operate the traditional marker-based motion capture system in which the errors can be fixed by using the temporal continuity of a point trajectory, assuming rigid bodies of participants' segments, and manually digitizing the correct position. This method may be appropriate for biomechanics researchers because the principles used are similar to those of the traditional marker-based approach. Another approach is to refine the neural network in the deep-learning algorithm. For example, in DeepLabCut (Nath et al., 2019), users evaluated the tracking results outputted from the first-trained neural network and perform secondary-training of the neural network to improve the performance of the algorithm. This method is appropriate for computer vision and machine learning researchers. Therefore, we utilized the first method to correct the tracking failure.

Other sources of error may be data processing, such as time synchronization. The error in the movement direction (i.e., Y direction for walking task and Z direction for jumping task), especially in the 30 Hz measurement (4K condition), tends to be large (Table 1). Within the time series profiles, the timing of synchronization appears to affect to the error of the two motion captures (Figure 3D). However, because this is a problem caused by the process of comparing the two motion captures, the effect of this error on the accuracy of the markerless system measurement should be relatively small.

We used the 1K (1, 920 × 1, 080 pixels at 120 Hz) and 4K (3, 840 × 2, 160 pixels at 30 Hz) conditions when using video-camera-based motion capture, which have lower sampling rates than those of the marker-based motion capture system. However, computer-vision-based pose estimation algorithms have the potential to extend the possibilities of measurement at high sampling rates exceeding 1,000 Hz. When measuring with current marker-based motion capture cameras at a sampling rate of 1,000 Hz or more, the angle of the camera view becomes very small, making it difficult to measure fast movements. Because a computer-vision-based pose estimation algorithm, such as OpenPose, can be applied to high-speed camera images, it is expected to overcome the difficulty.

Furthermore, we used five video cameras for markerless motion capture. Because, in the DLT method, the 3D position coordinates were calculated using the least squares method based on the joint coordinates in each camera coordinate system, the stability of the estimated 3D joint position improves as the number of cameras increases (i.e., the robustness against the probabilistic perturbation of the estimated joint position increases). In addition, if the number of cameras is sufficiently large, the camera that produces large tracking errors in a frame can be excluded from the 3D pose calculation. However, when manual processing is required for camera images, the processing cost increases proportionally with the number of cameras. Therefore, if a researcher increases the number of video cameras for a markerless motion capture, it is better to use the devices and programs that can automatically process most of the data.

In this study, we evaluated the accuracy of markerless motion capture through comparison with an optical marker-based motion capture. However, the marker-based system also has errors, which are mainly due to the skin artifact, (i.e., an error of joint position owing to the deformation of the skin on which the markers were attached on). The errors of the marker-based system can affect the validity of accuracy evaluation of markerless motion capture. Despite this limitation, the marker-based system has been recognized as the gold standard in the field of biomechanics. In addition, it is also difficult to make a similar comparison using a skinless cadaver because the cadaveric-based evaluation has limitations in movement. Therefore, our method for evaluating a markerless motion capture can be considered to be reasonable.

This study is preliminary work and thus requires further examination. Two perspectives may be important as a next step. First, the accuracy of joint positions could be improved so that other biomechanical parameters such as joint angle, joint angular velocity, and joint torque can be used. Thereafter, the accuracy of these biomechanical parameters should be investigated. Second, the task and aim of the study could be chosen so that the analysis can be performed within the accuracy of the current measurement system. Even if the accuracy of a markerless measurement is lower than that of the marker-based system, the markerless system has the advantage of environmental constraints. While limitations still exist, deep-learning-based markerless motion capture is expected to be applied in the future to sporting games and rehabilitations, which are considered to be difficult to measure with marker-based motion capture.

## Data Availability Statement

The raw data supporting the conclusions of this article will be made available by the authors, without undue reservation, to any qualified researcher.

## Ethics Statement

The studies involving human participants were reviewed and approved by Ethics Committee of the Graduate School of Arts and Sciences of the University of Tokyo. The patients/participants provided their written informed consent to participate in this study.

## Author Contributions

NN, TS, KU, and SY conceived and designed the work. NN, LO, AK, YI, SF, and SY performed the experiments. NN, TS, and SY analyzed the data. NN and SY interpreted the results. NN drafted the manuscript. TS, KU, LO, AK, YI, SF, and SY revised the manuscript.

## Conflict of Interest

The authors declare that the research was conducted in the absence of any commercial or financial relationships that could be construed as a potential conflict of interest.

## Supplementary Material

Supplementary Videos 1–3 The videos show the human pose during walking, jumping, and throwing obtained by two different motion capture.
